# Resource Allocation in 5G Cellular IoT Systems with Early Transmissions at the Random Access Phase

**DOI:** 10.3390/s25072264

**Published:** 2025-04-03

**Authors:** Anastasia Daraseliya, Eduard Sopin, Vyacheslav Begishev, Yevgeni Koucheryavy, Konstantin Samouylov

**Affiliations:** 1 Telecommunications Research Institute, HSE University, Moscow 101000, Russia; esopin@hse.ru (E.S.); vbegishev@hse.ru (V.B.); ykoucheryavy@hse.ru (Y.K.); ksamujlov@hse.ru (K.S.); 2 Probability Theory and Cybersecurity Department, Peoples’ Friendship University of Russia (RUDN University), Moscow 117198, Russia

**Keywords:** 5G, mMTC, CIoT, random access, early transmissions, delay, optimal resource allocation

## Abstract

While the market for massive machine type communications (mMTC) is evolving at an unprecedented pace, the standardization bodies, including 3GPP, are lagging behind with standardization of truly 5G-grade cellular Internet-of-Things (CIoT) systems. As an intermediate solution, an early data transmission mechanisms encapsulating the data into the preambles has been recently proposed for 4G/5G Narrowband IoT (NB-IoT) technology. This mechanism is also expected to become a part of future CIoT systems. The aim of this paper is to propose a model for CIoT systems with and without early transmission functionality and assess the optimal distribution of resources at the random access and data transmission phases. To this end, the developed model captures both phases explicitly as well as different traffic composition in downlink and uplink directions. Our numerical results demonstrate that the use of early transmission functionality allows one to drastically decrease the delay of uplink packets by up to 20–40%, even in presence of downlink traffic sharing the same set of resources. However, it also affects the optimal share of resources allocated for random access and data transmission phases. As a result, the optimal performance of 5G mMTC technologies with or without early transmission mode can only be attained if the dynamic resource allocation is implemented.

## 1. Introduction

Current 5G and future 6G systems need to offer a wide range of services, including enhanced mobile broadband, ultra-reliable low latency, and massive machine type communications (mMTC) [1]. While the former is already supported by 5G systems, the latter two services are lagging behind. At the same time, the market for the cellular Internet of Things (CIoT) is evolving at an unprecedented rate. In addition to conventional use-cases such as remote measurements, new services related to various industrial sectors such as electricity and oil distribution systems start to appear [2,3]. These services not only add to the already large deployment base of end devices (ED) but are generally characterized by much stricter requirements on delay and loss performance than mMTC [4].

Compared to conventional services offered by 5G mMTC systems such as Narrowband IoT (NB-IoT) and LTE-M technologies, 6G systems shall support various types of services in uplink and downlink directions. These include not only asynchronous transmission from inherently independent EDs but also software updates in downlink directions for smart EDs. To enable such versatility, 6G mMTC systems have to employ flexible frame structures with different and dynamically changeable allocations in uplink and downlink directions.

To enable a long-lasting lifetime for mMTC EDs that would still constitute the major traffic load in modern and future mMTC systems, they have to rely upon the random access (RA) phase followed by the data transmission (DT) phase. The RA phase is conventionally organized by utilizing the multichannel slotted ALOHA-type algorithms [5]. The DT phase relied upon multiplexing of uplink and downlink traffic over the orthogonal-frequency division multiple access (OFDMA) scheme. Depending on the traffic type and direction, ED is required to be served at both phases or only at the DT phase.

The use of RA and DT phases consecutively for small data transmissions brings significant signaling overhead to the system design. To this end, a number of optimizations have been proposed by 3GPP in the recent releases. One of the principal enhancements introduced in Release 16 is the use of early data transmissions (EDT) for small data [6]. This feature allows to transmission small data already at the RA phase. As highlighted in [7], under specific conditions, the use of EDT allows one to maximize the system’s capacity.

The aim of this paper is to propose a model of the service process in 5G/6G mMTC systems by explicitly accounting for design specifics of RA and DT phases, including the EDT operational regime and traffic service specifics in uplink and downlink directions. To this end, we formulate the system model as a multi-stage queuing system with multichannel ALOHA at the RA phase and a queuing system with batch arrivals at the DT phase. The latter allows us to explicitly account for traffic impact consisting of asynchronous transmissions in the uplink direction and software updates in the downlink one. We establish the stability conditions and then proceed to investigate user- and system-centric performance metrics including delay and system throughput.

The main contributions of our study are:

- A mathematical model for performance analysis of 6G CIoT mMTC systems accounting for service specifics at both RA and DT phases and different types of traffic at the air interface in uplink and downlink direction;
- Stability conditions of the considered system corresponding to the maximum system throughout and the associated delta of message transmission;
- Numerical results illustrating that: (i) the modern CIoT systems with fixed amount of resources allocated at the random access and data transmission phases are not optimized for different load conditions, (ii) flexible allocation allows one to reduce the latency of message transmission by a few times, and (iii) EDT operational regime results in consistently 20–40% reduction in the message transmission latency as compared to the traditional transmission scheme.

The paper is organized as follows. In Section 2, we discuss the related work. The system model is introduced in Section 3. We evaluate the formulated system for performance metrics of interest in Section 4. Numerical results are provided in Section 5. Finally, conclusions are presented in Section 6.

## 2. Related Work

Most studies where the authors addressed mMTC using CIoT technologies primarily focused on the RA phase. Since modern mMTC technologies, including both NB-IoT and LTE-M, utilize multichannel ALOHA mechanisms at the RA phase, most of the studies investigated them in detail under different traffic arrival patterns. The authors in [5] showed that, for multichannel slotted ALOHA under Poisson arrivals, the optimal transmission probability is inversely proportional to $\min(l/N_{t},1)$, where *l* is the number of preambles, and $N_{t}$ is the number of active EDs. The studies in [8,9] proposed a method for approximating the value of $N_{t}$. The NB-IoT RA phase performance under batch arrivals was addressed in [4]. Among others, the authors demonstrated that, under such types of arrival, capacity of the NB-IoT systems decreased by multiple orders of magnitude. A mixture of stochastic and regular arrivals was addressed in [10], in which the authors derived the mean delay for both traffic types. Unlike models of other types of traffic, such as enhanced Mobile BroadBand (eMBB) broadband traffic or Ultra Reliable Low Latency Communications (URLLC) traffic [11], RA mechanisms play a key role in the context of machine-to-machine (M2M) communications.

In addition, various algorithms have been developed for the RA phase to adaptively adjust access its parameters based on estimating the time-varying number of access requests [12,13,14,15,16]. In [13,14], an analytical model is presented to determine the expected total delay at the RA phase. Specifically, the mean number of non-conflicting preambles allocated by ED was reported in [12], the mean number of preambles experiencing collisions—in [13], and the mean number of EDs that successfully complete the RA phase—in [14]. The studies in [15,16,17,18] proposed the queuing theoretic models incorporating the queuing behavior of individual EDs. Another important characteristic in addition to transmission delay is the age of the information (AoI), which is investigated in [19,20,21].

Having identified that the NB-IoT and LTE-M RA designs are suitable for achieving high capacity at the RA phase for asynchronous EDs, researchers started to characterize the capacity at the DT phase under different deployments. The authors in [22] considered the optimal DT phase capacity for a given arrival rate of EDs having different resource requirements. Joint service deployment utilizing LTE and NB-IoT for surveillance applications was considered in [23].

Studies accounting for both the RA and DT phases have appeared only recently. Specifically, such systems were addressed in [24] for IEEE 802.16 [25] systems and, more recently, in [26,27] in the context of general random access systems. Specifically, the authors in [27] extended the model originally proposed in [24] and developed a method for determining the optimal ratio of resources at RA and DT phases to maximize the system throughput. Coexistence between mMTC and human-to-human (H2H) services was considered in [28], where the authors explicitly accounted for RA and DT phases for mMTC traffic and DT phase only—for H2H traffic. As mMTC technology bounds the ED battery lifetime, in [29], the authors explore the possibility that choosing among several low-power wide-area network (LPWAN) technologies integrated at a single ED may improve its lifetime.

An early data transmission regime (EDT) for the NB-IoT system introduced in 3GPP Release 16 [6] has received significant attention thus far. Specifically, the authors in [30] utilized computer simulations to show that, under careful choice of early and normal transmission probability, the capacity of the NB-IoT system can be maximized. The authors in [7] investigated the use of EDT operation in industrial environments, showing that it can enhance the system capacity compared to the traditional operational regime. However, no studies published so far addressed EDT performance when resources allocated to both the RA and DT phases may vary.

The review above illustrates that there are no studies jointly considering the RA and DT performance of mMTC systems having two phases with modern intelligent EDs that not only report the state of the remote system to the control system but are capable of receiving software updates over the downlink channel. Furthermore, there are no studies addressing performance of the EDT mechanism under different resource allocations to the RA and DT phases. In this paper, we will fill this void.

## 3. System Model

In this section, we describe the proposed system model. Although we mostly concentrate on the NB-IoT system in this study, we formulate it for a general setting such that the model is suitable for 5G mMTC systems that will utilize early transmission functionality as well.

### 3.1. Deployment and Resources

**Figure 1:**
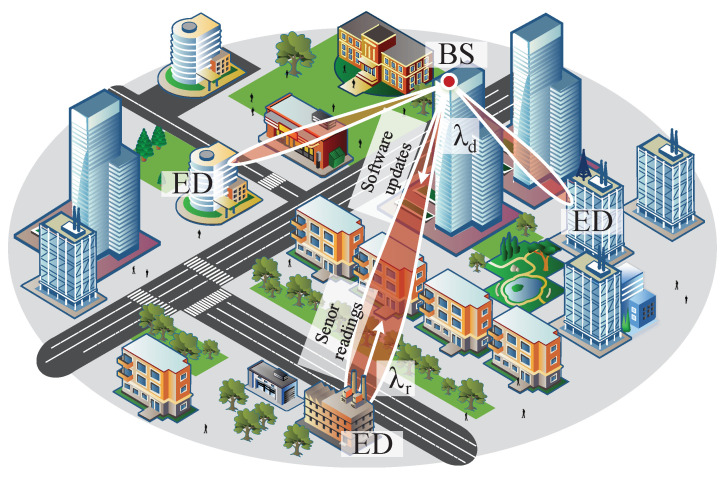
The considered mMTC deployment with downlink and uplink traffic for an early data transmission operation.

**Figure 2:**
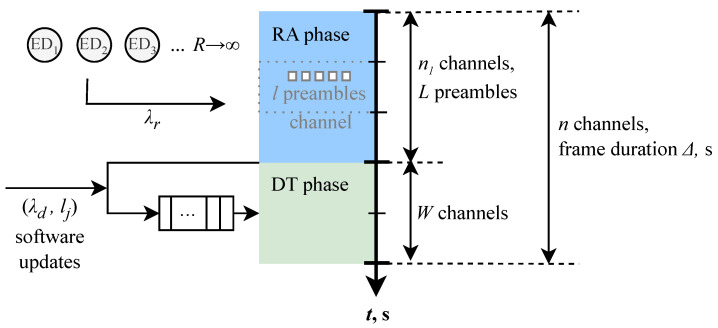
The frame structure, resources, and packet passing through RA and DT phases in the considered 5G mMTC system.

We consider a single base station (BS) serving end devices (ED) located in its coverage and requiring communications in both uplink and downlink directions; see Figure 1. Orthogonal frequency-division multiple access (OFDMA) is assumed. The available bandwidth is *B* Hz. The system operates in terms of frames with frame duration of Δms. The frame is divided into two parts—random access (RA) and data transmission (DT) phase—as shown in Figure 2. The time–frequency resources are measured in *n* channels (resource units, RU), where *n* is calculated according to the Nyquist theorem, n=Δ/(1/2B)=2Δ. Thus, out of *n* RUs, $n_{1}=\Delta_{1}/\left(1/2B_{1}\right)=2\Delta_{1}B_{1}$ RUs are allocated to the RA phase, whereas the remaining $W=n-n_{1}$ RUs are assigned to the DT phase. A single RU corresponds to the time required to transmit a single packet.

### 3.2. Service Process in Uplink and Downlink

EDs are associated with traffic in both the uplink and downlink directions. The service of a packet in the uplink direction requires the successful passing of RA only or RA and DT phase depending on the packet size; see Figure 2. If the packet size *S* is smaller than a certain threshold $T_{S}$, an early transmission mode is utilized, where the information is transmitted as part of the preamble. Otherwise, data are transmitted during the DT phase. It should be noted that uplink transmissions require one RU during the DT phase.

Traffic in the downlink direction proceeds directly to the DT phase, as the identifier of the ED to whom traffic needs to be sent is available at the BS. Both downlink and uplink traffic is multiplexed in the DT phase. At the RA phase, there are $n_{1}$ RUs, each having *l* preambles for the RA for uplink EDs. We denote $L=ln_{1}$. The packets in the DT phase are served by using the first come first served (FCFS) service discipline.

**Figure 3:**
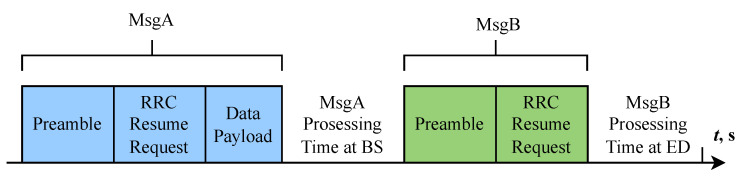
Illustration of two-step RA EDT scheme.

We consider and compare two access schemes: one that utilizes EDT and one that does not. In contrast to the four-step RA scheme specified in 3GPP Release 17 [31], in which each ED must initially transmit Msg1 to secure an uplink grant for the transmission of Msg3, the two-step RA scheme enables each ED to convey both Msg1 and Msg3 in a single message, designated as MsgA, without the necessity of an uplink grant. Consequently, the two-step RA scheme aligns with grant-free random access, whereas the four-step RA-SDT scheme corresponds to grant-based random access. As Figure 3 [15] illustrates, each node transmits its data payload along with the preamble in MsgA. If the random access response (RAR) message and the radio resource control (RRC) release message are received within the MsgB response window, then the node knows that its MsgA transmission has been successful.

### 3.3. Traffic Types and Models

All EDs are assumed to be sensory equipment. They operate asynchronously in the uplink direction. By following ITU-R M.2410 [32], we assume that new packets from these EDs arrive according to a Poisson process with an intensity $\lambda_{r}$. The packet size distribution is assumed to follow the probability mass function (pms) $f_{S}\left(k\right)$, k=0,1,…,K, where the argument is measured in bytes. By utilizing $T_{S}$ and $f_{S}\left(k\right)$, the probability that the early transmission mode is utilized is

(1) $$\begin{array}{c} p_{S}^{\left(E\right)}=\sum_{k=0}^{T_{S}-1}f_{S}\left(k\right), \end{array}$$

and with complementary probability, $\left(1-p_{S}^{\left(E\right)}\right)$, both RA and DT phases are needed for service.

Downlink traffic represents the software updates. We model this traffic using a bursty Poisson flow with intensity $\lambda_{d}$. We assume that the number of batches arriving in a single slot is

(2) $$\begin{array}{c} \gamma_{k}=\frac{{\left(\lambda_{d}\Delta \right)}^{k}}{k!}e^{-\lambda_{d}\Delta }. \end{array}$$

and each batch consists of $\left\{l_{k}\right\}$ packets proceeding directly to the DT phase. We denote the mean batch size by $\bar{l}$.

### 3.4. Metrics of Interest

In our paper, we are interested in the optimal resource division between the RA and DT phases such that the mean delay is minimized. To achieve this goal, we characterized the stability conditions of the system and then derived the mean packet delay in the uplink and downlink directions. We utilize latency as the main performance, as it is defined as the major quality of service indicator for mMTC services in ITU-R M.2410 [32] and ITU-R M.2412 [33]. To account for the system performance, we also characterized throughput corresponding to the optimal division of resources as the main operator-side performance metric.

We specifically note that the considered in this paper EDT regime allows one to additionally save power at NB-IoT EDs. It is enabled by keeping the ED transceiver up for shorter periods of time. However, the ultimate impact is linear and for this reason we omit it here.

## 4. Performance Evaluation Framework

In this section, we establish the analytical framework. We begin by formalizing the Markov chain model of the system and then proceed to define the stability conditions. Subsequently, we divide the model into two sub-models and analyze them separately. Finally, we derive the metrics of interest.

### 4.1. Two-Dimensional Markov Chain Model

The model described in the previous section can be represented by a two-dimensional Markov chain $X_{n}=\left(R_{n},D_{n}\right)$. Here, $R_{n}$ is the number of active EDs in the RA phase, and $D_{n}$ is the number of packets queued in the DT phase in the *n*th frame. We now parameterize this process by providing the transition probabilities between states.

As the new active EDs arrive according to a Poisson process with intensity $\lambda_{r}$, the number of active EDs in the RA phase at each frame follows a Poisson distribution with parameter $\lambda_{r}\Delta$, where Δ is the frame duration. We denote by $\alpha_{k}$, k=0,1,…, the probability that *k* new EDs arrive within a single frame.

The number of EDs that successfully passed the RA phase depends on the number of active EDs in the frame. Assume that the system is in state (r,d). Hence, the probability $\beta_{k}\left(r\right)$ that *k* out of *r* EDs will successfully pass the RA phase is provided in [12]:

(3) βk(r)=1LrLk∑i=0min(L−k,r−k)(−1)iL−kir!(r−k−i)!(L−k−i)r−k−i,0≤k≤min(L,r),

implying that the mean number B(L,r) of EDs that successfully pass the RA phase in a single frame with *r* active EDs and *L* preambles is calculated as

(4) $$\begin{array}{c} B\left(L,r\right)=r{\left(\frac{L-1}{L}\right)}^{r-1}. \end{array}$$

**Figure 4:**
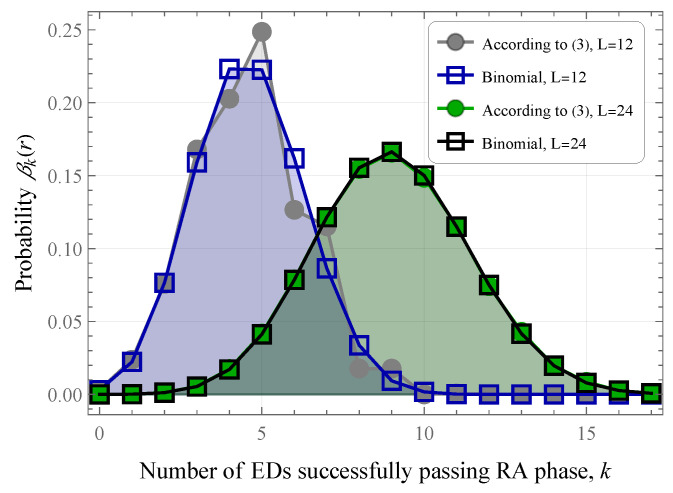
Approximation of (3) by binomial distribution.

The state-space of the system for realistic values of the number of preambles *L* and the number of active EDs is expected to be large; we propose to approximate probabilities $\beta_{k}\left(r\right)$ by a binomial distribution with parameters m=min(L,r), and p=B(L,r)/m. This approximation is accurate even for small values of *L* and improves as *L* increases, as shown in Figure 4. With these parameters, we ensure that the expected number of EDs passing the RA phase in a time slot coincides with the initial distribution for all values of *L* and *r*. In addition, it can be shown that variances of the initial distribution and the binomial distribution with the specified parameters converge to the same value for r=L and large values of *L*.

By accounting for batch arrivals in the downlink direction in the DT phase and for the number of EDs that successfully pass the RA phase, we can derive the transition probability from state (r,d) to state (s,g) in the most general cases as

(5) $$\begin{array}{cc} p_{\left(r,d),(s,g\right)} & =\sum_{i=0}^{\min(L,r)}\beta_{i}\left(r\right)\alpha_{s-r+i}\sum_{j=0}^{g-d-i-W}\gamma_{j}l_{g-d-i-W}^{\left(j\right)}. \end{array}$$

### 4.2. Stability and Maximum Throughput

One of the critical measures for the system of interest is the maximum throughput that can be attained. However, systems with random access and unlimited population of EDs are not always stable. Thus, to determine the maximum throughput, we first need to determine the stability conditions of the introduced system model.

We note that the Markov chain $R_{n}$ that describes the number of active EDs on the RA phase is not ergodic in the strict mathematical sense. This is because the expected number of EDs B(L,r) that successfully complete the RA phase in a single frame tends to zero as the number of active EDs increases. Therefore, if there is a constant average arrival rate, the Markov chain cannot be considered stable.

On the other hand, it can be derived from expression (4) that B(L,r) has a maximum. Indeed, following [12], we see

(6) $$\begin{array}{c} \frac{B(L,r+1)}{B(L,r)}=\left\{\begin{array}{cc} >1, & r<L-1,\\ =1, & r=L-1,\\ <1, & r>L-1. \end{array}\right. \end{array}$$

From (6), we immediately see that the mean number of EDs passing the RA phase reaches a maximum at r=L−1 and r=L. Thus, when the mean number of new ED arrivals to the RA phase is less than the maximum B(L,L), the considered system may be called metastable, as it spends quite a long time in states with the number of active EDs near *L* before going to infinity. Using this argument, we establish the following necessary (but not sufficient) conditions for metastability:

(7) $$\begin{array}{c} \lambda_{r}\Delta <B\left(L,L\right). \end{array}$$

For large numbers of preambles *L*, this condition can be approximated by

(8) $$\begin{array}{c} \lambda_{r}\Delta <Le^{-1}, \end{array}$$

which is in agreement with previously known asymptotics, e.g., [5].

Note that (7) is related to the RA phase. For the overall system to be metastable, in addition to (7), we also need to ensure that the mean arrival rate to the DT phase is less than the maximum number of transmitted packets *W*, resulting in the following second metastability condition:

(9) $$\begin{array}{c} (\lambda_{r}+\lambda_{d}\bar{l})\Delta <W. \end{array}$$

### 4.3. Decomposed Model

As shown in Section 4.2, the Markov chain $R_{n}$ that describes the number of active EDs in the RA phase is not ergodic. As a result, the stationary distribution of $R_{n}$ does not exist, making the numerical analysis impossible. To avoid this, we first propose artificially limiting the maximum states of the system at the RA and DT phases using large values *R* and *D*. These values should be empirically chosen such that packet losses are negligible. Second, we decompose the two-stage service model into separate systems associated with the RA and DT phases. For the considered system, it is feasible because the number of active EDs in the RA phase, $R_{n}$, is independent of the number of EDs in the DT phase, $D_{n}$. The resulting Markov chains capturing the dynamics of the RA and DT phases are one-dimensional, making them simpler to analyze.

#### 4.3.1. RA Phase Model

**Figure 5:**
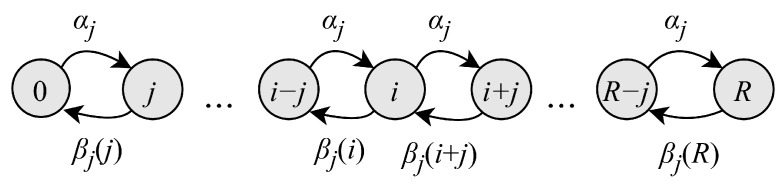
State transition diagram of the RA phase model.

Let $R_{n}$ denote the number of active EDs in the RA phase. The number of active EDs at this phase can be represented by a Markov chain, as illustrated in Figure 5. The transition probabilities of this process, $p_{i,j}^{\left(R\right)}=P\left\{R_{n+1}=j|R_{n}=i\right\}$, take the form

(10) $$\begin{array}{cc} \; & p_{i,j}^{\left(R\right)}=\;\sum_{k=\max(0,i-j)}^{i}\;\beta_{k}\left(i\right)\alpha_{j-i+k},0\leq i\leq R,\;0\leq j<R,\\ \; & p_{i,R}^{\left(R\right)}=\sum_{k=0}^{i}\beta_{k}\left(i\right)\left(1-\;\sum_{s=0}^{R-i+k-1}\;\alpha_{s}\right),\;0\leq i\leq R. \end{array}$$

The stationary probabilities $\{q_{r}\},r=0,1,\ldots ,R$ are given by the solution of the system of equilibrium equations, which can be derived using the transition probabilities

(11) $$\begin{array}{cc} \; & q_{r}^{\left(R\right)}=\sum_{i=0}^{R}q_{i}^{\left(R\right)}p_{i,r}^{\left(R\right)},\;0\leq r\leq R. \end{array}$$

The system in (11) is solved numerically to obtain the stationary probabilities of $R_{n}$, which, in turn, allow the derivation of the probability $\theta_{k}$ that *k* EDs successfully pass the RA phase and move to the DT phase in one frame. Each ED independently of the others remains in the RA phase with probability $p_{S}^{\left(E\right)}$ for early transmission, and with probability $1-p_{S}^{\left(E\right)}$ it goes to the DT phase. Let there be *i* independent elections, where each ED has an equal chance of entering the DT phase independent of the others. If there are *i* such EDs, the probability that *k* of them will switch to the DT phase follows a binomial distribution. Then, the probability $\theta_{k}$ can be calculated as

(12) θk=∑j=kR∑i=kmin(j,L)ikpS(E)i−k1−pS(E)kqj(R)βi(j),0≤k≤L.

We specifically note that, in the case when early transmission regime is not utilized, that is, $p_{S}^{\left(E\right)}=0$, (12) simplifies to the following form:

(13) $$\begin{array}{c} \theta_{k}=\sum_{j=k}^{R}q_{j}^{\left(R\right)}\beta_{k}\left(j\right),\;0\leq k\leq L. \end{array}$$

The main metric of interest in the RA phase is the mean number of EDs that successfully pass through the RA phase and move to the DT phase. This is immediately given by

(14) $$\begin{array}{c} \bar{\theta }=\sum_{k=0}^{L}k\theta_{k}. \end{array}$$

#### 4.3.2. DT Phase Model

**Figure 6:**
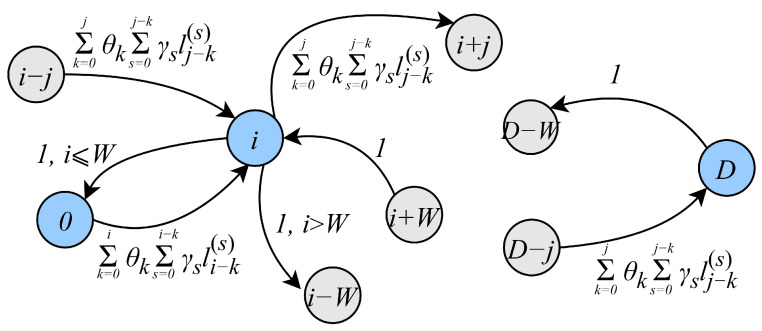
A part of the transition diagram of the DT phase model.

Consider the Markov chain $D_{n}$ representing the packet service process in the DT phase. At each frame, there are *k* packets arriving from the RA phase with probability $\theta_{k}$ and *i* batches of packets arriving directly to the DT phase with probability $\gamma_{i}$. Recall that the number of packets in a batch is distributed according to the pmf $\left\{l_{j}\right\}$. However, note that, at most, *W* packets can be transmitted in each frame. Consequently, the transition probabilities $p_{i,j}^{\left(D\right)}=P\left\{D_{n+1}=j|D_{n}=i\right\}$ take the form of (15), where $l_{i}^{\left(s\right)}$ is the probability that *s* batches contain *i* packets, and are evaluated as *s*-fold convolution of the initial distribution $\left\{l_{j}\right\}$. A part of the state transition diagram of the DT phase Markov model with associated transitions is shown in Figure 6.

(15) $$\begin{array}{cc} \; & p_{i,j}^{\left(D\right)}=\sum_{k=0}^{j}\theta_{k}\sum_{s=0}^{j-k}\gamma_{s}l_{j-k}^{\left(s\right)},\;0\leq i<W,\;0\leq j<D,\\ \; & p_{i,D}^{\left(D\right)}=\sum_{k=0}^{L}\theta_{k}\left(1-\sum_{s=0}^{D-k-1}\gamma_{s}\sum_{r=0}^{D-k-1}l_{r}^{\left(s\right)}\right),\;0\leq i<W,\\ \; & p_{i,j}^{\left(D\right)}=\;\sum_{k=0}^{j-i+W}\theta_{k}\sum_{s=0}^{j-i+W-k}\gamma_{s}l_{j-i+W-k}^{\left(s\right)},\;W\leq i\leq D,\;i-W\leq j<D,\\ \; & p_{i,D}^{\left(D\right)}=\sum_{k=0}^{L}\theta_{k}\left(1-\sum_{s=0}^{D-i-k+W-1}\gamma_{s}\sum_{r=0}^{D-i-k+W-1}l_{r}^{\left(s\right)}\right).\;W\leq i\leq D. \end{array}$$

Once the transition probabilities have been deduced, the equilibrium equations for the system can be written in the following form:

(16) $$\begin{array}{c} q_{k}^{\left(D\right)}=\sum_{j=0}^{k+W}q_{j}^{\left(D\right)}p_{j,k}^{\left(D\right)},\;0\leq k\leq D, \end{array}$$

which can be solved numerically for stationary probabilities.

### 4.4. Metrics of Interest

Once the models representing the dynamics of the RA and DT phases are solved, the desired performance metrics can be estimated. Recall that the most important metric for the considered system is the mean delay. To obtain this, we used Little’s law. According to it, the mean number of EDs in the RA phase is given by

(17) $$\begin{array}{c} \bar{R}=\sum_{k=1}^{R}kq_{k}^{\left(R\right)}. \end{array}$$

Similarly, the mean number of packets queued at the DT phase is provided by

(18) $$\begin{array}{c} \bar{D}=\sum_{k=1}^{D}kq_{k}^{\left(D\right)}. \end{array}$$

As the loss probabilities are negligible, the mean delay for the uplink packet can be expressed as

(19) $$\begin{array}{cc} w_{r} & =p_{S}^{\left(E\right)}\frac{\bar{R}}{\lambda_{r}}+\left(1-p_{S}^{\left(E\right)}\right)\left(\frac{\bar{R}}{\lambda_{r}}+\frac{\bar{D}}{\bar{\theta }+\lambda_{d}\bar{l}\Delta }\Delta \right). \end{array}$$

It can be observed that the downlink packets experience delay in the DT phase only. For these packets, the mean delay is given by

(20) $$\begin{array}{c} w_{d}=\frac{\bar{D}}{\bar{\theta }+\lambda_{d}\bar{l}\Delta }\Delta . \end{array}$$

The upper bound of the maximum throughput $C_{R}$ on the RA phase can be evaluated directly from (7) as

(21) $$\begin{array}{c} C_{R}=\frac{L}{\Delta e}, \end{array}$$

leading to the following maximum throughput $C_{D}$ of the DT phase

(22) $$\begin{array}{c} C_{D}=\frac{W}{\Delta }-\lambda_{r}. \end{array}$$

Finally, the utilization *U* of the DT phase is

(23) $$\begin{array}{c} U=\frac{1}{W}\sum_{k=1}^{W-1}kq_{k}^{\left(D\right)}+\sum_{k=W}^{D}q_{k}^{\left(D\right)}. \end{array}$$

## 5. Numerical Analysis

In this section, we numerically elaborate the proposed framework. Specifically, we first utilize the developed model to assess performance metrics when the resources at the RA and DT phases are fixed, e.g., as it is done in modern CIoT technologies such as LTE-M and NB-IoT while EDT functionality is not utilized. Then, we will show how the system capacity can be improved by utilizing flexible RA/DT resource allocation. Finally, we demonstrate how the optimal resource allocations changes when EDT functionality is enabled.

The default system parameters are provided in Table 1. To compute these metrics, we utilized NB-IoT numerology and assumed the use of a single resource block (RB) of 180 KHz. The RU is selected such that it allows transmission of a single 128 bytes packet.

### 5.1. Fixed RA/DT Resource Allocation

**Figure 7:**
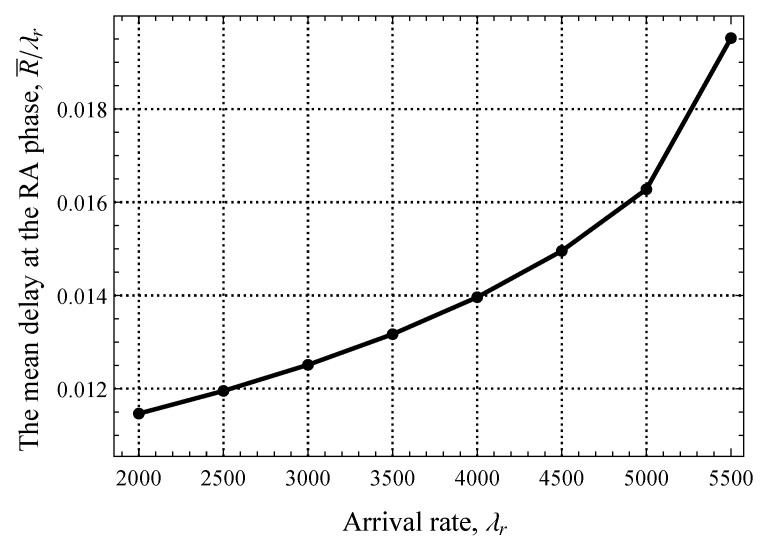
The mean delay at the RA phase.

We begin by reporting the performance of the system where the amount of resources allocated for the RA and DT phases is fixed and no EDT regime is utilized. To this end, we start with the delay at the RA phase illustrated in Figure 7, where only uplink packets are present. Note that here and in what follows, we show the results for the stable system conditions that are assessed by utilizing the criterion provided in Section 4. Here, we observe a typical behavior of a system with decentralized access in stable conditions—as the arrival rate of EDs generating traffic in the uplink direction, $\lambda_{r}$, increases, the mean delay increases exponentially. We specifically attract attention to the fact that, for a wide range of values of $\lambda_{r}$, the mean delay stays extremely low and changes insignificantly, by just 6 ms. This implicitly highlights that, even for $\lambda_{r}=5500$ pkts/s, the RA phase remains underloaded.

**Figure 8:**
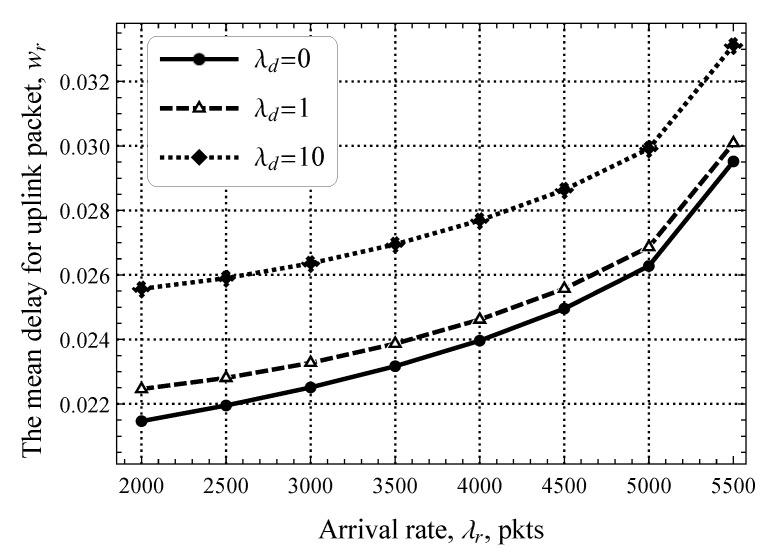
The mean delay for uplink packets.

We now show the full mean delay experienced by uplink packets including both the RA and DT phases illustrated in Figure 8, where the values of the software update intensity $\lambda_{d}$ are chosen such that the offered traffic load is 0% ($\lambda_{d}=0$ upd./s) and ≈3% of the DT utilization ($\lambda_{d}=1$ upd./s) and ≈30% ($\lambda_{d}=10$ upd./s). By analyzing the results in Figure 8, we see that the presence of downlink traffic at the DT phase does not significantly contribute to the overall delay of uplink traffic. In fact, the difference between $\lambda_{d}=0$ upd./s and $\lambda_{d}=1$ upd./s is less than 1 ms and increases by 3–4 ms for $\lambda_{d}=10$ upd./s. Furthermore, the gap between curves corresponding to different values of $\lambda_{d}$ becomes smaller as the intensity of uplink traffic increases. This implies that not only the DT phase in modern CIoT technologies may handle the additional load but this phase is severely overprovisioned in absence of software update traffic. As a result, to make mMTC CoI technologies scalable for diverse traffic load conditions, the amount of resource allocation to the RA and DT phases needs to be dynamically adjusted.

### 5.2. Optimal RA/DT Resource Allocation

Having observed that the static allocation of resources to the RA and DT phases may lead to under-utilization of the DT phase, we now proceed to assess what would be the optimal division between them resulting in the maximal throughput.

**Figure 9:**
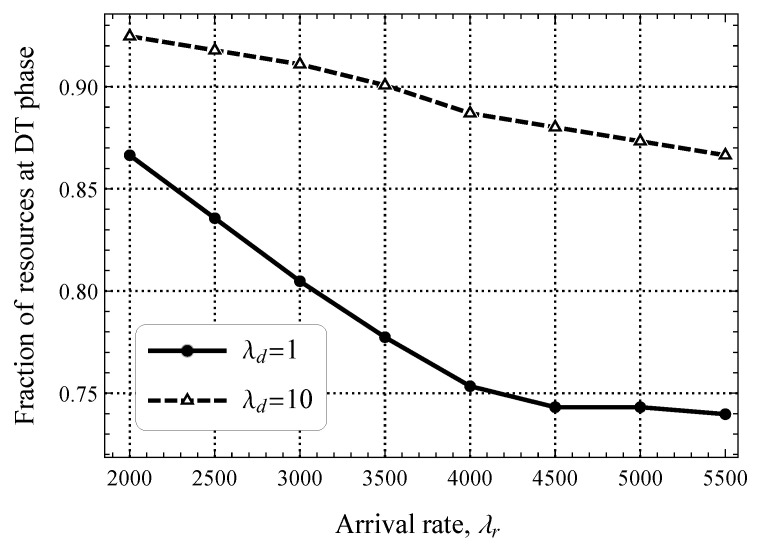
Fraction of resources allocated for the DT phase.

Figure 9 shows the optimal fraction of resources (in terms of the minimal mean uplink delay) that need to be allocated to the DT phase as a function of $\lambda_{r}$ and two software update intensities, $\lambda_{d}=1$ upd./s and $\lambda_{d}=10$ upd./s. As one may observe, the optimal division of resources varies drastically depending on the arrival rate of packets in the uplink and downlink directions. The current allocation utilized in 4G/5G CIoT systems is closer to the case when the DT phase is highly loaded with software update traffic ($\lambda_{d}=10$ upd./s, ≈30% of DT utilization). For more realistic loads in the downlink direction, the capacity of the DT phase must be kept smaller to accommodate more packets in the uplink direction as the RA phase becomes a bottleneck.

### 5.3. Performance with EDT Enabled

**Figure 10:**
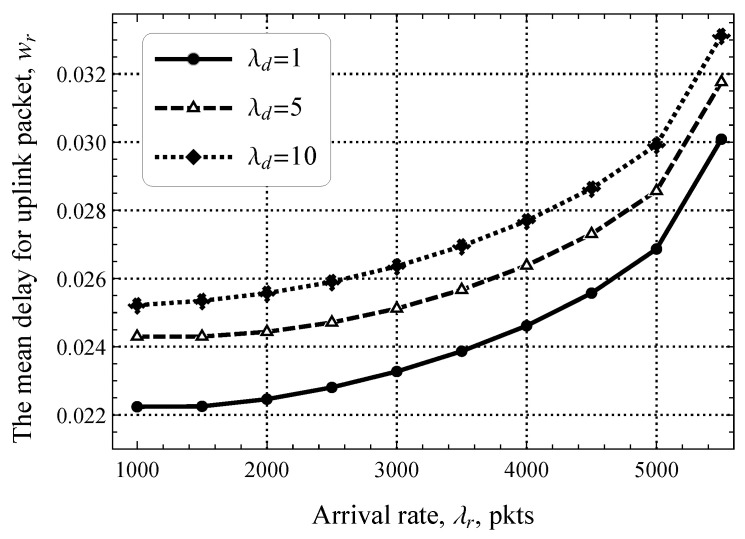
The mean delay for uplink packets as a function of $\lambda_{d}$.

Finally, we assess the performance when the EDT regime is enabled. We begin with the delay of packets in the uplink direction $w_{r}$. To this end, Figure 10 illustrates the study metric as a function of the rate of software updates in the downlink direction, where $T_{S}$ is set to 0, that is, all uplink packets require service at both the RA and DT phases for $\lambda_{d}=1,5,10$ upd/s. As one may observe, the least delay is attained for the lowest load in the downlink direction, i.e., $\lambda_{d}=1$ upd/s. When this rate increases, the delay increases linearly. The rationale is that uplink packets share the same DT phase as downlink packets. It can be observed that the delay also increases as $\lambda_{r}$ increases but the difference between the different curves becomes smaller. The rationale is that, for high values of $\lambda_{r}$, more load is imposed in the DT phase and the impact of the RA phase on the overall delay of the uplink packets becomes smaller.

**Figure 11:**
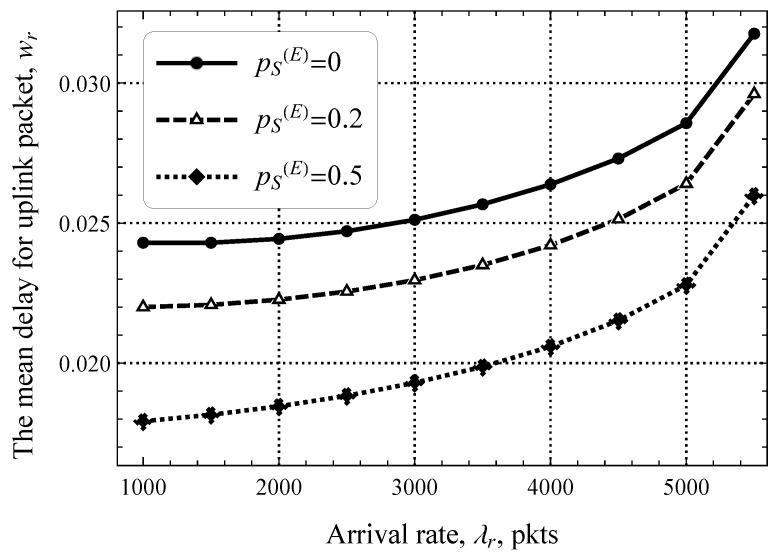
The mean delay for uplink packets as a function of $p_{S}^{\left(E\right)}$.

Furthermore, Figure 11 shows the packet delay in the uplink direction as a function of the packet size threshold, $p_{S}^{\left(E\right)}$, for a fixed value of $\lambda_{d}=5$ upd/s. Here, we can see that the impact of $p_{S}^{\left(E\right)}$ is significant. Specifically, when half of the packets can already be transmitted in the RA phase, the delay decreases by 20–40%. This gap also decreases as $\lambda_{d}$ increases but is still noticeable. By comparing these results to those shown in Figure 10, we see that the system with $p_{S}^{\left(E\right)}=0.5$ and $\lambda_{d}=5$ upd/s performs twice better than the one with $p_{S}^{\left(E\right)}=0$ and $\lambda_{d}=10$ upd/s. This behavior shows that early transmission is a useful mechanism for mMTC systems.

**Figure 12:**
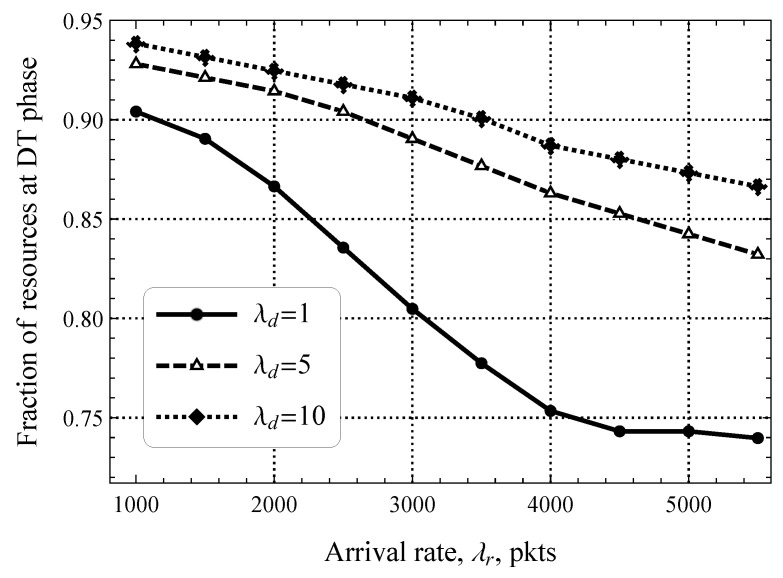
Fraction of resources allocated for the DT phase.

**Figure 13:**
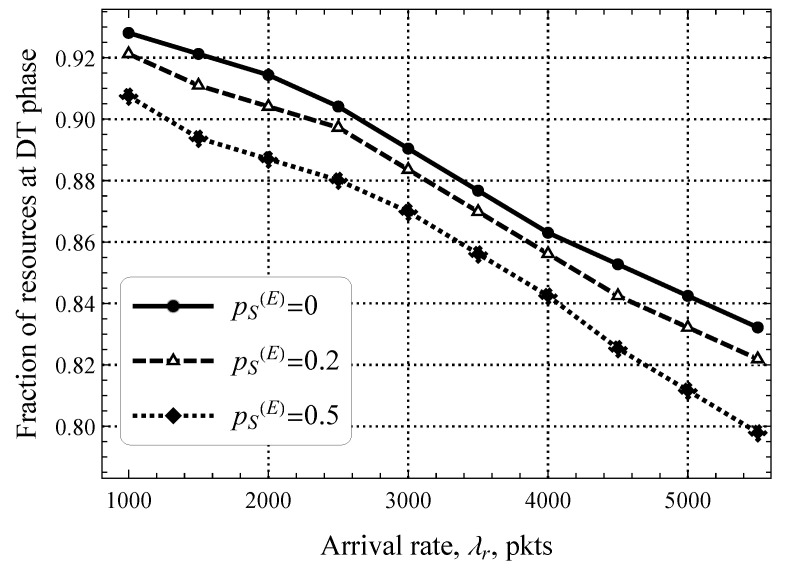
Fraction of resources allocated for the DT phase.

In previous illustrations, we have seen that both the rate of software updates in the downlink direction $\lambda_{d}$ and the packet size threshold $p_{S}^{\left(E\right)}$ significantly affect uplink packet delay. However, these illustrations were prepared by assuming a fixed distribution of resources between the RA and DT phases. Now, we demonstrate the optimal allocation of these resources as a function of the parameters $\lambda_{d}$ and $p_{S}^{\left(E\right)}$ in Figure 12 and Figure 13, respectively. Starting with the former, plotted for different $\lambda_{d}$ and fixed $p_{S}^{\left(E\right)}=0$, we observe that the optimal fraction of resources allocated for the DT phase varies drastically, that is, the range of values is 0.75–0.95, which deviates significantly from the default division. Getting deeper into details, we highlight that the joint impact of $\lambda_{d}$ and $\lambda_{r}$ is important. Specifically, for small values of $\lambda_{d}$, the difference between the fraction of resources that must be allocated for the DT phase between $\lambda_{d}=1$ upd/s and $\lambda_{d}=10$ upd/s is just 0.04. However, for $\lambda_{r}=5000$, it was already 0.13.

The impact of different values of $p_{S}^{\left(E\right)}$ on the optimal fraction of resources allocated to the DT phase shown in Figure 13 for $\lambda_{d}=5$ upd/s is more straightforward. Here, we logically see that as $\lambda_{r}$ increases, the study metric also decreases linearly. The rationale is that early transmission affects the RA phase significantly unloading the DT phase, which heavily contributes to the delay under high traffic conditions. In general, the presented results show that the optimal performance of 5G mMTC technologies with or without early transmission mode can only be attained if dynamic resource allocation between the RA and DT phases is feasible.

### 5.4. Comparison of NB-IoT Transmission Schemes

**Figure 14:**
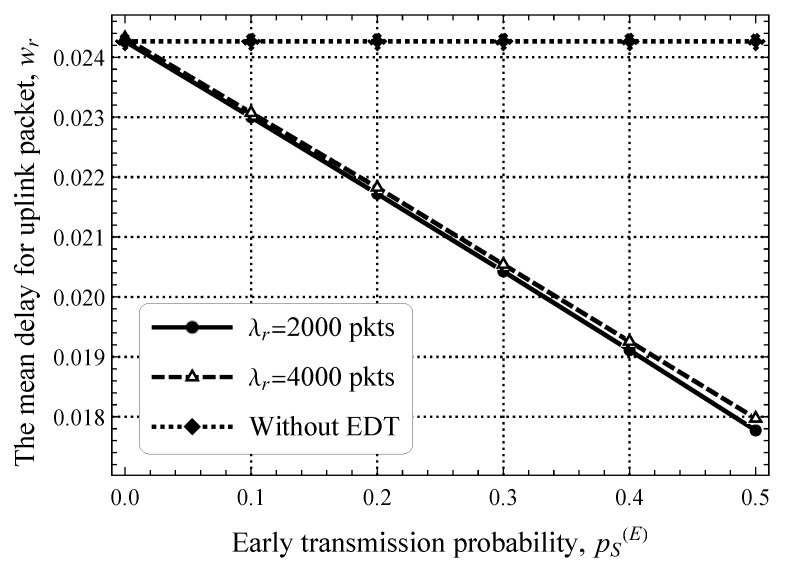
The mean delay for uplink packets for optimal resource allocations between the RA and DT phases.

In this section, we provide a direct comparison of NB-IoT transmission schemes in terms of delay. To this end, Figure 14 shows the mean delay for uplink packets for optimal resource allocations between the RA and DT phases. Confirming the expected predictions, the gain relative to the transmission delay increases when more users transmit small amounts of data. Specifically, for $p_{s}^{\left(E\right)}=0.2$, the delay gain is already almost 3 ms, while for $p_{s}^{\left(E\right)}=0.4$, it raises further to 6 ms, which results in an approximately 20–40% delay reduction.

By cross comparing the results presented for conventional access and for EDT schemes, we can also observe that, for the load point that optimally divides the resources between the RA and DT phases, the EDT gain in latency is rather consistent and is around 20–40% correctly observed. This is mainly because EDT allows one to skip DT phase, leaving only the RA phase loaded.

## 6. Conclusions

Motivated by the introduction of the early transmission regime in NB-IoT technology, in this study, we developed a model that allows the assessment of delay performance in 5G mMTC systems with uplink and downlink traffic and various resource allocation at the RA and DT phases. The developed model was further utilized to evaluate the optimal resource allocations between the random access and data transmission phases such that uplink delay is minimized.

Our results demonstrate that the use of an early transmission regime allows a drastic decrease in the delay of uplink packets in 5G mMTC systems. However, the optimal usage of this functionality requires careful optimization of the resources allocated to the random access and data transmission phases. The latter needs to be performed dynamically depending on the packet size distribution of EDs in the uplink direction and can be implemented by utilizing the proposed framework.

In our study, we considered bursty traffic in the downlink direction corresponding only to software updates. In principle, the model can be extended to capture the case of uplink correlated behavior of EDs that is caused, e.g., by external control of end devices by upper layer protocols such as SCADA. This can be done by utilizing the methods recently proposed in [4,10]. This is especially interesting in the context of optimal division of resources between RA and DT phases considered in our study. The reason is that the studies in [4,10] demonstrated that the capacity of a single NB-IoT call under standardized ITU-R M.2412 traffic conditions (one message per 2 h from a single ED) decreases from multiple thousands of EDs to just 800–100 EDs when performance guarantees of ITU-R M.2410 are met. In our future studies, we will consider the case of bursty traffic conditions in the uplink direction. Another interesting research direction is comparison of the optimal resource allocation reported in this paper to the two-way handshake operation that was recently introduced in [15].

## Figures and Tables

**Table 1:** Default system parameters.

Notation	Description	Value
*B*	Bandwidth	180 KHz
Δ	Frame duration	0.01 s
*l*	Number of preambles	12
*P*	RU (packet) size in uplink direction	128 bytes
*W*	Number of RU at the DT phase	278 RUs
$n_{1}$	Number of RU at the RA phase	14 RUs
*n*	Overall number of RU in a frame	292 RUs
$\lambda_{r}$	Uplink packet arrival rate	2–5.5K pkts/s
$\lambda_{d}$	Rate of software updates	1, 5, 10 upd/s
$\left\{\gamma_{k}\right\}$	Number of updates in a slot	Poisson
$\left\{l_{j}\right\}$	pmf of the software update batch	Binomial
$\bar{l}$	Mean software update size in RUs	100/500 RUs
$p_{S}^{\left(E\right)}$	Early transmission probability	0, 0.2, 0.5

## Data Availability

Data are contained within the article.
